# Engraftment of enteric neural progenitor cells into the injured adult brain

**DOI:** 10.1186/s12868-016-0238-y

**Published:** 2016-01-25

**Authors:** Jaime Belkind-Gerson, Ryo Hotta, Michael Whalen, Naema Nayyar, Nandor Nagy, Lily Cheng, Aaron Zuckerman, Allan M. Goldstein, Jorg Dietrich

**Affiliations:** Department of Pediatrics, Massachusetts General Hospital, Harvard Medical School, Boston, MA 02114 USA; Department of Pediatric Surgery, Massachusetts General Hospital, Harvard Medical School, Boston, MA 02114 USA; Department of Neurology, Division of Neuro-Oncology, and Center for Regenerative Medicine, Massachusetts General Hospital, Harvard Medical School, Boston, MA 02114 USA; Pediatric Neurogastroenterology Program, Massachusetts General Hospital, Harvard Medical School, 175 Cambridge St #575, Boston, MA 02114 USA

**Keywords:** Enteric neuronal progenitor cells, Stem cells, Brain injury, Cell transplantation, Brain repair

## Abstract

**Background:**

A major area of unmet need is the development of strategies to restore neuronal network systems and to recover brain function in patients with neurological disease. The use of cell-based therapies remains an attractive approach, but its application has been challenging due to the lack of suitable cell sources, ethical concerns, and immune-mediated tissue rejection. We propose an innovative approach that utilizes gut-derived neural tissue for cell-based therapies following focal or diffuse central nervous system injury.

**Results:**

Enteric neuronal stem and progenitor cells, able to differentiate into neuronal and glial lineages, were isolated from the postnatal enteric nervous system and propagated in vitro. Gut-derived neural progenitors, genetically engineered to express fluorescent proteins, were transplanted into the injured brain of adult mice. Using different models of brain injury in combination with either local or systemic cell delivery, we show that transplanted enteric neuronal progenitor cells survive, proliferate, and differentiate into neuronal and glial lineages in vivo. Moreover, transplanted cells migrate extensively along neuronal pathways and appear to modulate the local microenvironment to stimulate endogenous neurogenesis.

**Conclusions:**

Our findings suggest that enteric nervous system derived cells represent a potential source for tissue regeneration in the central nervous system. Further studies are needed to validate these findings and to explore whether autologous gut-derived cell transplantation into the injured brain can result in functional neurologic recovery.

**Electronic supplementary material:**

The online version of this article (doi:10.1186/s12868-016-0238-y) contains supplementary material, which is available to authorized users.

## Background

Diseases of the nervous system belong to the most challenging maladies and have been recognized as one of the greatest threats to public health [1–5]. With a current lack of effective therapies for most neurological disorders, such as neurodegenerative diseases, traumatic brain injury, or long-term neurotoxicity from cancer therapy, and a steadily growing aging population with increasing prevalence of neurological disorders, there is an urgent need to develop strategies for nervous system repair.

Neural plasticity and nervous system regeneration in the setting of injury or disease is dependent on the activity of neural stem and progenitor cells that reside in a specialized, regulatory neurovascular environment [6–11]. Optimizing the cellular and molecular factors that control the neural stem cell niche has evolved as an attractive strategy to promote central nervous system (CNS) repair following focal and diffuse brain injury [12]. However, the damaged central nervous system has a limited capacity for endogenous regeneration. Therefore, exogenous stem cell-based therapies remain a promising avenue to promote tissue and functional repair [13–15]. Experimental studies designed to transplant exogenous neural cells into the injured CNS have largely failed due to multiple reasons, including technical and ethical factors regarding the source of donor cells, immune-mediated transplant rejection, and insufficient integration of transplanted cells into the host cellular network [16, 17].

Identifying an easily accessible, autologous, and reliable source of neural progenitor cells would address many of the current challenges and might be useful in the treatment of a wide range of neurologic disorders. The objective of this study was to test the hypothesis that neural progenitor cells derived from the enteric nervous system (ENS) could have a potential role in CNS repair. We and others have previously shown that enteric neuronal stem and progenitor cells (ENSCs) can be successfully isolated from the postnatal ENS, propagated in vitro, and transplanted into the gut in vivo [18–20]. Building on this knowledge, we transplanted gut-derived enteric neural progenitor cells into brain-injured mice.

We demonstrate that following both systemic and local administration, transplanted cells (1) home to neurogenic niches, (2) survive and differentiate into neuronal and glial lineages, and are able to (3) stimulate endogenous neurogenesis. These results support the potential use of autologous gut-derived neural tissue for cell replacement therapy in the injured CNS.

## Methods

### Animals

The Institutional Animal Care and Use Committee of the Massachusetts General Hospital approved all experiments. We used two strains of genetically engineered animals as the source of donor cells to facilitate their in vivo tracking based on the expression of fluoresecent proteins: Actb-DsRed mice (strain Tg(CAG-DsRed*MST)1Nagy/J; Jackson Labs Stock #005441) were purchased from the Jackson Laboratory. All cells in Actb-DsRed mice are fluorescently labeled [21]. We also used transgenic Nestin-GFP mice, in which GFP expression is controlled under the Nestin promoter [22]. Nestin-GFP mice were used as donor mice initially, but in light of better breeding efficiency, we used Actb-DsRed donor mice in later experiments. Recipient mice were wild-type C57BL/6.

### Isolation and propagation of ENSCs in vitro

Mice were sacrificed on postnatal day 14-21 (P14-21), and the gastrointestinal tract from duodenum to anus was removed. The longitudinal muscle-myenteric plexus (LMMP) layer was separated and dissociated with dispase (250 μg/ml; StemCell Technologies, Vancouver, Canada) and collagenase XI (1 mg/ml; Sigma Aldrich, St. Louis, MO, USA) at 37 °C for 1 h. The cell suspension was passed through a 40μm cell strainer and cultured at a density of 50,000 cells/mL in proliferation medium, consisting of Neurocult NSC Basal Medium (StemCell Technologies) supplemented with 20 ng/ml epidermal growth factor (EGF; StemCell Technologies) and 10 ng/ml basic fibroblast growth factor (bFGF; StemCell Technologies), 0.0002 % Heparin (StemCell Technologies) and 100U/ml Penicillin–Streptomycin (Life Technologies) for 7–10 days to form enteric neurospheres. Neurospheres were passaged every 7–10 days with gentle Accutase (StemCell Technologies) dissociation followed by re-plating in culture medium containing conditioned medium (1:2 mix). ENSCs retain the capacity to form neurospheres following up to five passages. For consistency, we used secondary neurospheres throughout this study.

To induce differentiation, neurospheres were dissociated with Accutase (StemCell Technologies) then passed though a 40 μm cell strainer and plated at 50,000 cells/mL on glass-bottom chamber slides coated with 20 mg/mL fibronectin (Biomedical Technologies, Ward Hill, MA, USA). Cells were cultured for 7 days in NeuroCult NSC Differentiation Medium (StemCell Technologies) prior to immunohistochemistry.

### Brain injury models

#### Closed head injury (concussion model)

Mice were injured daily for 5 consecutive days, and transplanted into mice 3 days after the last injury. During injury, mice were anesthetized with 4 % isoflurane for 45 s, grasped by the tail, and placed prone. The head of the animal was placed directly beneath the opening of a metal tube conduit. A 54-g metal bolt was dropped onto the midline of the head from a height of 38 inches. Sham-injured mice were subjected to anesthesia but no weight drop.

#### Radiation injury

Mice were treated with either focal brain or whole body irradiation. For focal brain irradiation, the mouse body was protected with a customized lead shield and irradiation administered to anesthetized mice as a single fraction of 10 Gy using a Cs-irradiation source. Whole body irradiation was performed using a sublethal dose of 5 Gy in a similar fashion.

### Cell transplantation

Prior to transplantation, dissociated ENSCs were pulsed for 2–4 h with 10 µM BrdU (3.3 µg/ml) to determine in vivo DNA synthesis as a surrogate marker for cell proliferation. For cell transplantation directly into the brain, 4–6 week-old wild-type mice were anesthetized with isoflurane and ENSCs (5000 cells per recipient) were stereotactically injected into various brain regions using a Kopf stereotaxic frame (David Kopf Instruments; Tujunga, CA, USA). Injected regions included the lateral subventricular zone (SVZ) (coordinates: AP 1.0 mm, ML 1.0 mm, DV 2.1 mm), the dentate gyrus (DG) of the hippocampus (coordinates: AP −1.70 mm, ML 0.70 mm, DV 2.04 mm), and the left lateral ventricle (LV) (coordinates: AP 0 mm, ML 1 mm, DV 2.5 mm). Systemic cell transplantation was performed via tail vein injection of ENSCs (100–400 k cells per recipient, depending on the experiment).

### Immunofluorescence

Cells or tissues were fixed, washed, and permeabelized with 0.1 % Triton X-100 for 30 min and then exposed to the following primary antibodies: mouse anti-Tuj1 (1:100, Covance, Dedham, MA, USA), mouse anti-Hu C/D (1:100, Life Technologies, Carlsbad, CA, USA), rabbit anti-p75 (1:500, Promega, Madison, WI, USA), rabbit anti-Sox2 (1:50, Abcam, Cambridge, MA, USA), rabbit anti-Doublecortin (1:250, Abcam), rabbit anti-Olig2 (1:250, Millipore, Billerica, MA), goat anti-GFAP (1:500, Abcam), goat anti-GFP (1:400, Rockland, Limerick, PA, USA). Secondary antibodies included goat anti-mouse IgG Alexa Fluor 546, goat anti-rabbit Alexa Fluor 488, donkey anti-goat Alexa Fluor 488, donkey anti-goat Alexa Fluor 546, donkey anti-mouse Alexa Fluor 546, donkey anti-mouse Alexa Fluor 488, all from Life Technologies (Carlsbad, CA, USA). Cell nuclei were stained with DAPI (Vector Labs, Burlingame, CA, USA).

### Confocal microscopy

Z-stack images were obtained with laser scanning confocal microscopy using a Nikon A1R confocal microscope, providing high-resolution images of up to 4096 × 4096 pixels with a galvano scanner.

## Results

### Postnatal gut-derived ENSCs can be isolated and propagated in culture

**Fig. 1:**
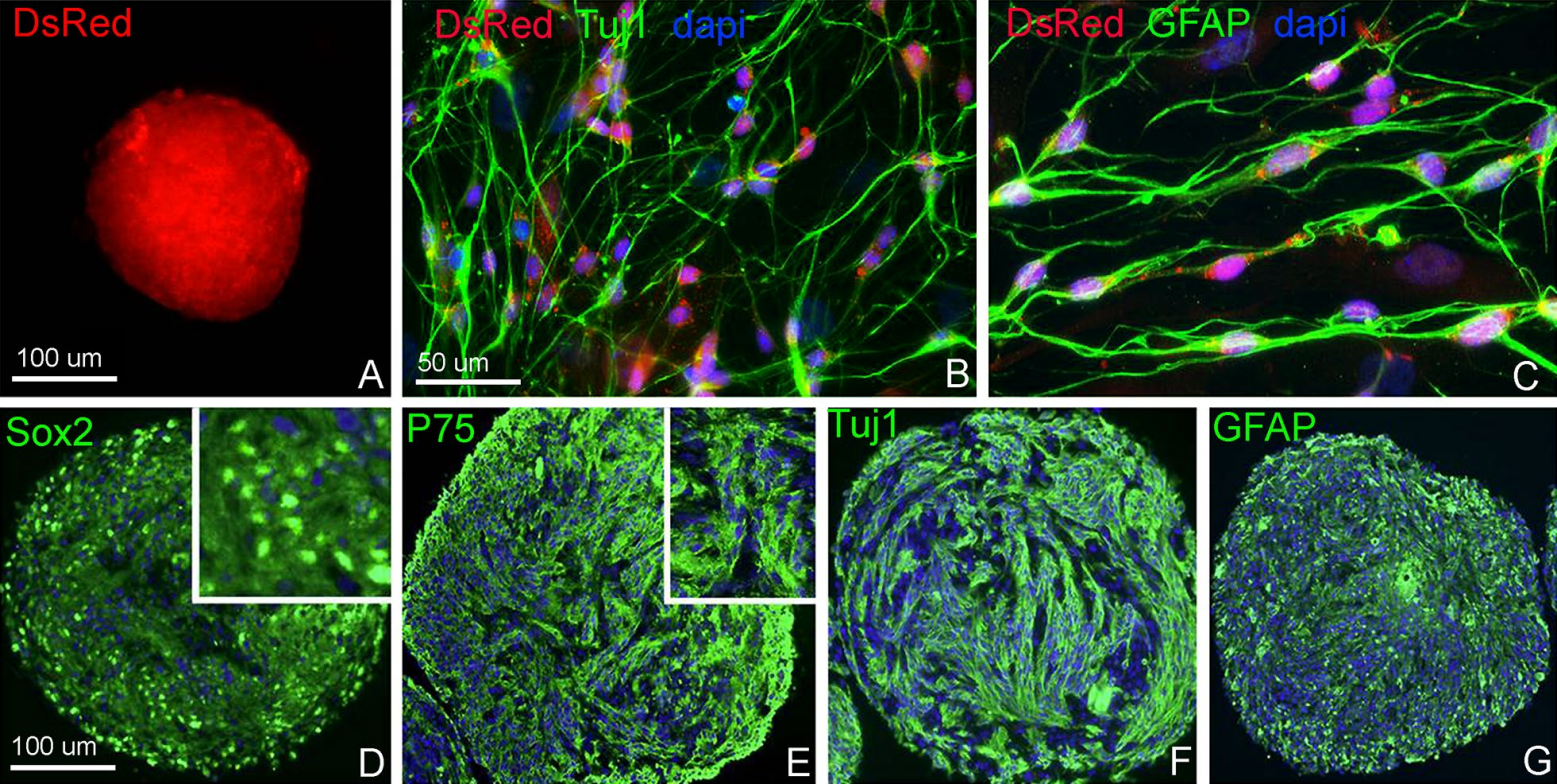
In vitro characterization of multipotent, self-renewing progenitor cells from the postnatal ENS. Enteric neurospheres derived from Actb-DsRed mouse colon express red fluorescent protein (**A**). Enteric neuronal stem/progenitor cells (ENSCs) within those neurospheres differentiate into neurons (**B**) and glia (**C**) that retain DsRed expression. Immunohistochemical characterization shows that cells within the neurospheres express markers of neural progenitors (Sox2, **D**; P75, **E**), enteric neurons (Tuj1, **F**), and glial cells (GFAP, **G**)

To determine whether ENSCs could serve as a potential source for CNS repair, the intestinal tract of 2–3 week-old postnatal Actb-DsRed mice was removed and ENSCs isolated and propagated, as previously described [23, 24]. ENSCs are able to form neurospheres in culture and these spheres can be labeled with markers for enteric neuronal progenitors (Sox2 and p75) as well as neuronal (Tuj1) and glial (GFAP) markers (Fig. 1). We have previously shown that enteric neurospheres contain 37.3 ± 4.2 %; neurons and 27.6 ± 1.7 glial cells (Hotta et al. in press). Dissociated neurospheres give rise to Tuj1 + and GFAP + cells, consistent with neuronal and glial differentiation, respectively (Fig. 1B, C, F, G).

### Following intracerebral injection ENSCs survive and stimulate endogenous neurogenesis

**Fig. 2:**
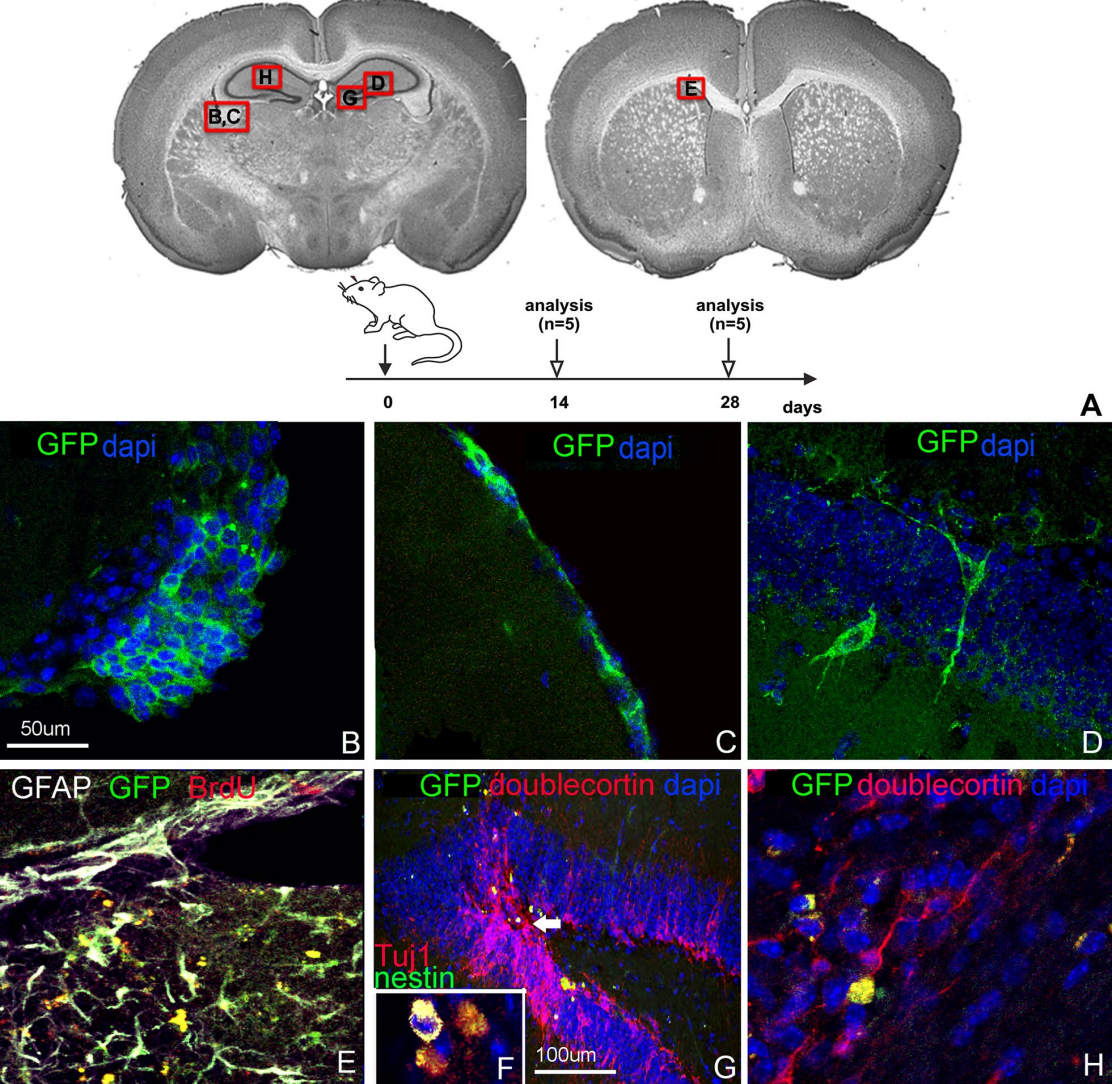
Survival and localization of ENSCs following intracerebral delivery. Nestin-GFP^+^ ENSCs were injected into various brain regions and mice were analyzed at 2 weeks and 4 weeks post transplantation into the brain regions depicted (**A**). Transplanted cells were found in clusters (**B**) and monolayers (**C**) along the ventricular lining, and within the parenchyma of the hippocampus formation (**D**, **F**–**H**) and subventricular zone (**E**). BrdU labeling confirms survival of proliferating ENSCs (**E**). Differentiation into glial (**E**) and neuronal (**F**–**H**) phenotypes is also seen (**F** represents an inset from the same region as **G**, with Tuj1 as a marker of mature neurons). Increased numbers of endogenous doublecortin^+^ neurons within the dentate gyrus are identified in close proximity to GFP^+^ transplanted cells (**G**, *arrow*)

To determine whether gut-derived neural progenitor cells can survive after transplantation into the adult brain, ENSCs were prepared, pulsed with BrdU in vitro, and injected as a cell suspension (5000 cells in 3 µl volume) into various brain regions (lateral subventricular zone, dentate gyrus of the hippocampus, and lateral ventricle). In this pilot experiment, Nestin-GFP-labeled cells were used [22] with subsequent experiments using Actb-DsRed-labeled cells. Mice were sacrificed after 2 and 4 weeks following transplantation (n = 5 per group). Immunohistochemistry identifies gut-derived GFP+ neural precursors in the brains of transplanted animals at both time points. Following injection into the lateral ventricle, cells were found along the inner lining of the ependymal layer of the ventricular system (Fig. 2B, C) and along the needle tract (not shown). GFP + cells were also seen in the brain parenchyma in and around the dentate gyrus following transplantation into the hippocampal region (Fig. 2D–G), where rare transplanted cells were identified to display a branched morphology with apparent integration into the granular cell layer of the dentate gyrus (Fig. 2D). This tissue integration was only observed at 4 weeks, but not at 2 weeks after transplantation.

Cells co-expressing both GFP and BrdU were identified, suggesting that proliferating ENSCs survive for at least 4 weeks after transplantation (Fig. 2E). A subpopulation of transplanted cells co-expressed doublecortin, a marker of immature neurons (Fig. 2F–H), and GFAP, a glial cell marker (Fig. 2E). Interestingly, enhanced endogenous neurogenesis, as evidenced by focally increased doublecortin expression, was observed in the granular cell layer of the dentate gyrus in close proximity to the location of the transplanted cells (Fig. 2G).

### ENSCs delivered systemically home to the injured brain

**Fig. 3:**
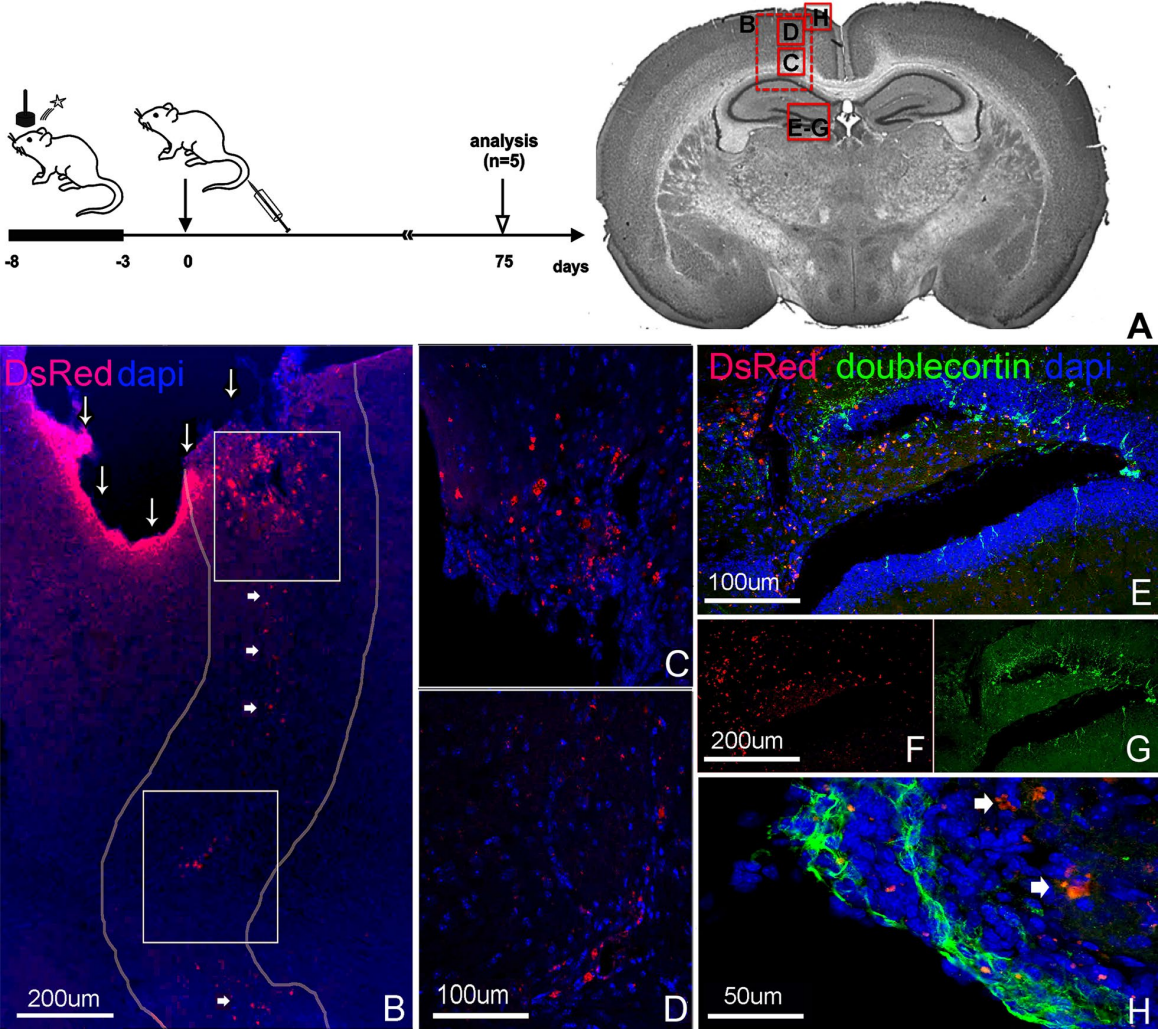
Following concussion injury, ENSCs cluster around injured brain parenchyma, migrate to neurogenic niches, and induce focal neurogenesis. Concussion injury was induced over 5 days, ENSCs delivered systemically 3 days later, and brains analyzed after 10 weeks. Brain regions shown in* each panel* are indicated in (**A**). ENSCs are identified at the site of brain injury, both at the cortical surface (**B**, *thin arrows*) and in the parenchyma (**B**–**G**). ENSCs appear to be migrating from the injury site toward the ipsilateral dentate gyrus (**B**, *thick arrows*). Doublecortin + endogenous neurons are observed in increased numbers in the region of the transplanted cells in the dentate gyrus (**E**–**G**) and at the cortical surface (**H**, *arrows* highlight transplanted ENSCs adjacent to endogenous doublecortin + neurons)

Based on our initial experiments employing cell delivery via intracerebral injection, we asked whether systemically delivered cells would be able to home to the sites of injury. We focused on clinically relevant injury models, including an injury model caused by concussion (closed head injury) and an injury model caused by irradiation. Following concussion injury, as described in Methods, Actb-DsRed + ENSCs were injected systemically via tail vein (400,000 cells in 200 µL; n = 3). Controls were injected with cell-free media (n = 2). All mice survived until sacrifice at 10 weeks post transplantation. Systemically administered ENSCs were identified 10 weeks post-transplantation. We found ENSCs homing to the injury site at the cortical surface, as shown in Fig. 3B. Transplanted cells were also identified within the brain parenchyma with apparent migration along white matter tracts (Fig. 3A–C), connecting the injury site with the *ipsilateral* dentate gyrus (Fig. 3B–G). A significant increase in resident doublecortin + cells, suggestive of increased endogenous neurogenesis, was observed predominantly in areas adjacent to where transplanted cells were identified (Fig. 3E–H). As shown in Fig. 3E, endogenous doublecortin expression was more pronounced in areas where DsRed + cells were located (upper part of the image, Fig. 3E), but not in areas devoid of DsRed + cells (lower part of the image, Fig. 3E). In this experiment, we were not able to detect DsRed + cells that co-labeled with doublecortin.

To determine whether systemically delivered cells also home to other organs, we analyzed lung tissue at 24 h and up to 75 days post-injection. DsRed + ENSCs were found in clusters in the lung at 24 h (Additional file 1: Figure S1), but were not seen at 75 days (not shown).

### Systemically delivered ENSCs are identified throughout the brain following radiation injury

Based on our observations we hypothesized that focal brain injury induced by either needle injury or concussion resulted in significant neural and vascular tissue damage, perhaps facilitating the entry of ENSCs into the brain parenchyma.

**Fig. 4:**
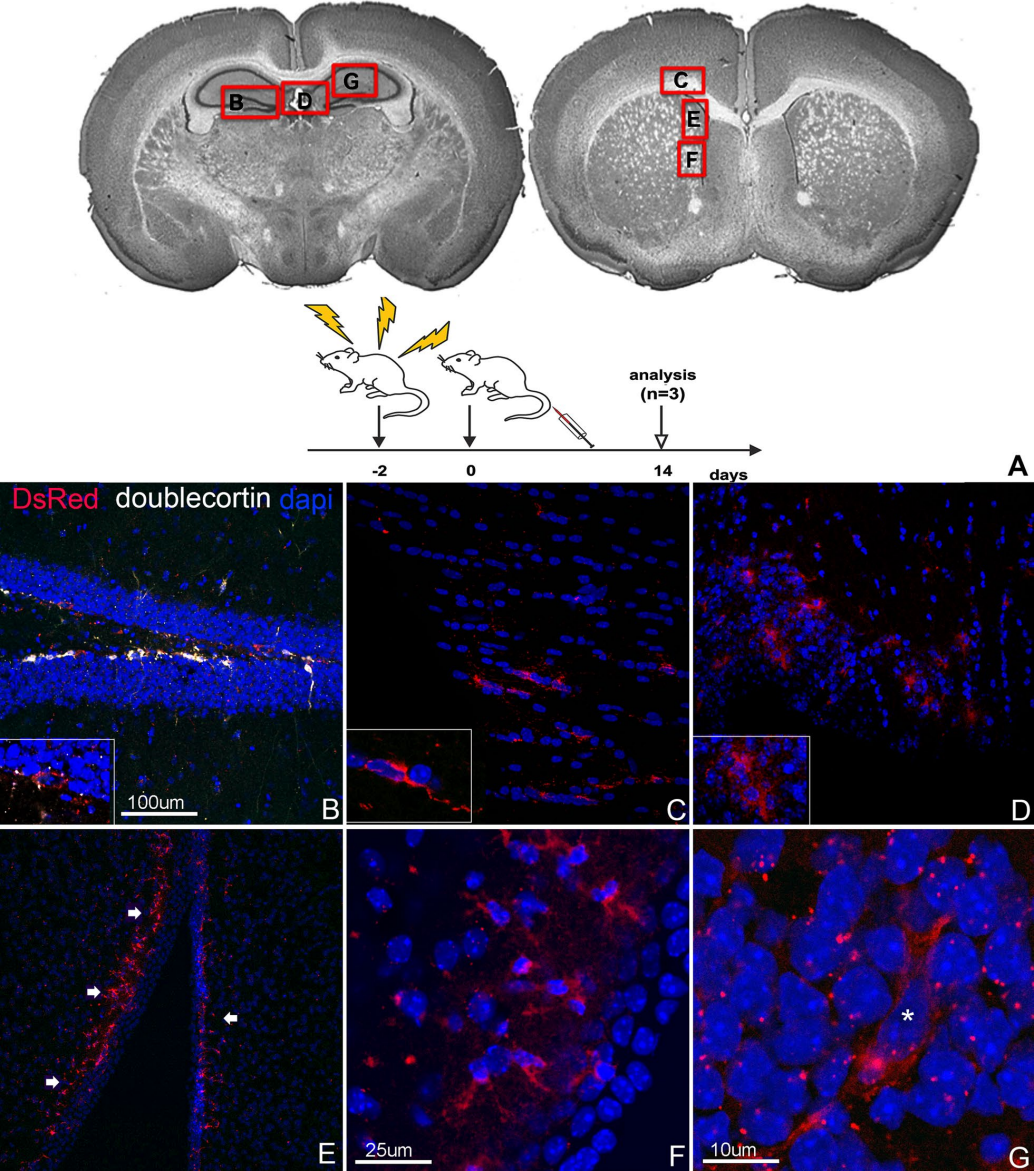
Systemically delivered ENSCs home to the brain following whole body radiation. ENSCs were delivered systemically 2 days after whole body radiation and mice analyzed at 14 days. Brain regions shown in each* panel* are indicated in (**A**). DsRed + cells are present in the subgranular layer of the dentate gyrus, alongside endogenous doublecortin + (*white*) neurons (**B**). Transplanted cells are also found within large white matter tracts bordering the subventricular zone (**C**), choroid plexus (**D**), and subependymal layer of the subventricular zone (**E**–**G**)

To explore whether systemically delivered ENSCs are able to enter the central nervous system in the setting of radiation injury, considered less traumatic to the integrity of brain tissue, we performed an experiment using a whole-body (including brain) radiation injury. A sub lethal radiation dose of 5 Gy was administered to mice and ENSCs (100,000 cells in 400 µL) delivered via tail vein 48 h later. Control animals were irradiated and treated with saline injection via tail vein. Animals were examined 14 days following cell delivery. Notably, DsRed + cells were identified in multiple brain regions, and were especially enriched in the germinal zones of the brain (SVZ and DG) and large white matter tracts. Specifically, cells were found in the granular cell layer of the dentate gyrus (Fig. 4B), the corpus callosum, one of the largest white matter tracts in the central nervous system (Fig. 4C), the choroid plexus, a highly vascularized tissue within the ventricular system (Fig. 4D), and the subependymal layer of the lateral ventricle (Fig. 4E–G).

**Fig. 5:**
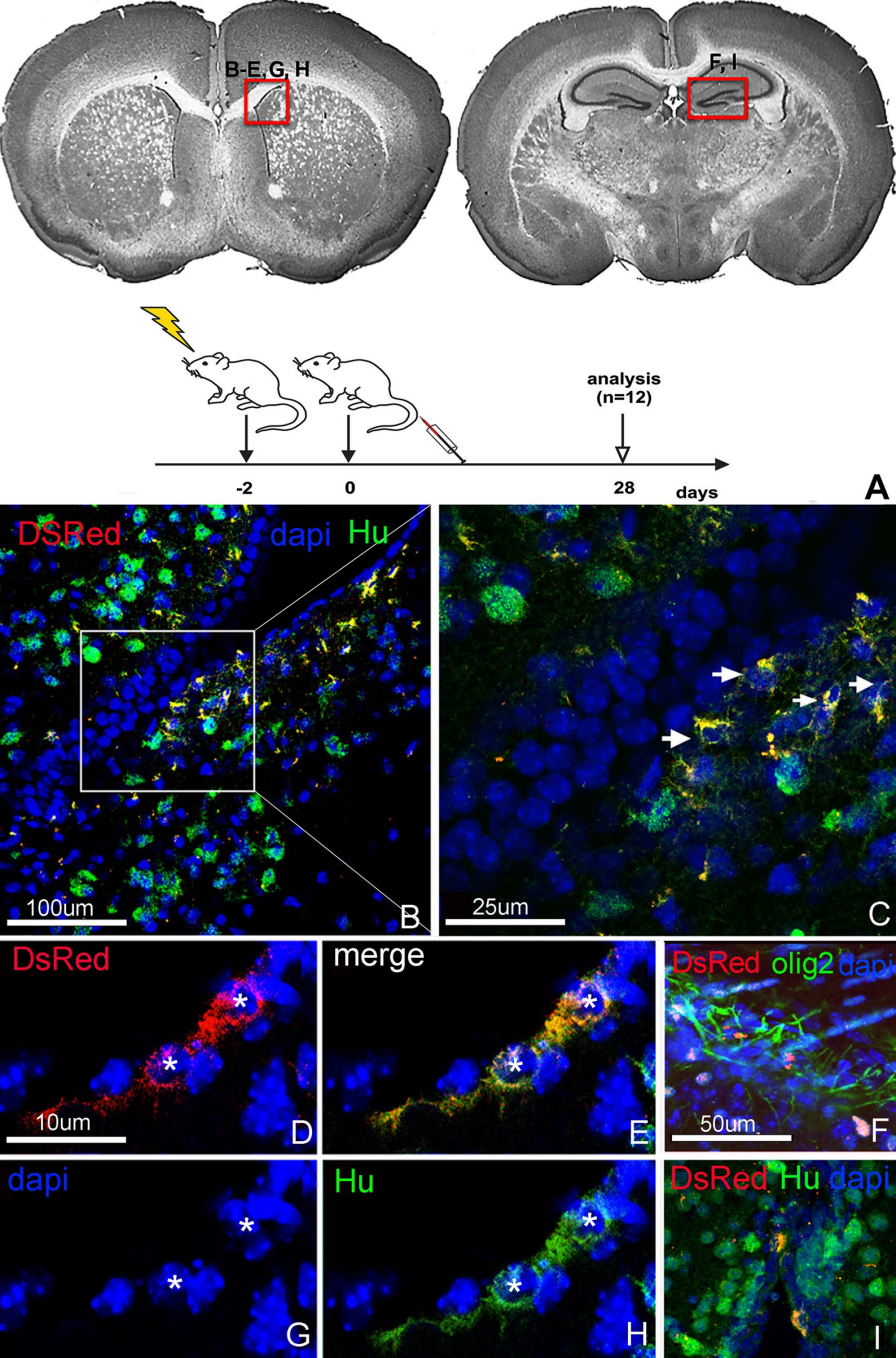
ENSCs home to the brain and undergo neuronal differentiation after focal brain irradiation. Mice were subjected to focal brain irradiation, transplanted with DsRed + ENSCs via tail vein 2 days later, and analyzed at 28 days. Brain regions shown in each* panel* are indicated in (**A**). Transplanted cells are present in the subventricular zone and co-express Hu, consistent with neuronal differentiation (**B**–**E**, **G**, **H**). DsRed + cells are also seen in the dentate gyrus (**I**), in close proximity to Olig2 + oligodendrocytes (**F**)

Based on the findings of this experiment, we modified our experimental setting to reflect a more relevant clinical scenario. As whole body irradiation is mainly reserved for patients treated with bone marrow transplantation and is less commonly used in clinical practice, we modified our experimental design to use focal brain irradiation instead, which is frequently applied to patients with brain cancer. The brain of mice was irradiated with 10 Gy in a single fraction and ENSCs (400,000 cells in 400 µL) were delivered via tail vein 48 h later. One experimental group (brain irradiation and ENSC delivery, n = 3) was compared to three control groups (irradiation without ENSCs, n = 3; no irradiation with ENSCs, n = 3; and no irradiation without ENSCs, n = 3). Mice were analyzed 28 days after cell delivery. The results confirmed our preliminary findings, showing the presence of transplanted cells in germinal zones, including the subependymal layer of the ventricular zone (Fig. 5B–D) and the dentate gyrus (Fig. 5I), as well as within white matter tracts (Fig. 5F). Interestingly, many of the transplanted cells identified in mice analyzed at 28 days co-labeled with anti-Hu, a mature neuronal marker. Notably, in non-irradiated animals, DsRed + cells were not readily detectable in the brain. We also did not identify the presence of intravenously-delivered ENSCs in the intestine, suggesting that tissue injury may be a prerequisite for the homing of systemically-delivered cells.

## Discussion

The main objective of this study was to determine whether neural progenitor cells derived from the ENS could survive in the adult mammalian brain after transplantation and could have a potential role in repair of the injured CNS. We found that transplanted ENSCs survive, proliferate, and differentiate into neuronal and glial lineages in vivo. Moreover, transplanted cells migrate extensively along pre-existing pathways and modulate the local microenvironment to stimulate endogenous neurogenesis.

We recently showed that Nestin-expressing neural progenitor cells can be isolated from the intestine and propagated in vitro to give rise to neurospheres capable of differentiating into neuronal and glial lineages [23]. These findings were confirmed in the present study. We have also previously shown that these neurospheres can generate enteric neurons upon transplantation into aneural embryonic hindgut [23]. Interestingly, brain- and gut-derived neurospheres appear to possess phenotypically similar features, including the ability to form dense neuroglial networks in culture and the expression of similar markers of pluripotency [23]. However, their functional properties following transplantation in vivo have not yet been compared. Based on our previous success in isolating, culturing, and transplanting gut-derived neurospheres, and their phenotypic similarities to brain-derived neurospheres, we initiated the present proof-of-principle study.

One of the current challenges compromising the success of cell transplantation strategies for the injured central nervous system has been immune rejection of transplanted cells by the host organism. A particular strength of the current approach was the use of enteric-derived neuronal progenitors, aiming at an elimination of detrimental immune rejection mechanisms if used in an autologous fashion. In addition, cell delivery to injured tissue typically relies on direct donor cell injection, an invasive procedure that results in local tissue damage and other morbidity [25, 26]. In a recent paper, ENS progenitor cells were injected into the hippocampus of irradiated mice, but cell survival was poor and no differentiation was observed [26]. Based on these findings, it remains unclear whether direct tissue injury and local inflammation with microglial activation caused by injection, as reported by Osman et al. [26], could hinder cell survival and integration into the host. In the present study, we tested the feasibility of *systemic delivery* to the injured nervous system. To further validate the clinical relevance and practicality of this approach, we used three different injury models, including needle injury, concussion injury, and brain irradiation.

Our findings suggest that gut-derived neural progenitor cells, genetically engineered to express fluorescent proteins, can be successfully transplanted into the injured adult brain. These cells home to sites of injury following systemic cell delivery. Transplanted cells were able to survive at least 10 weeks, proliferate as reflected by BrdU incorporation, and exhibited both neuronal and glial immunophenotypes in vivo [27]. The ENSCs we detected appeared to be anatomically integrated into the tissue as shown in Figs. 2 and 4, although their functional integration into the neuronal network remains unknown. We noted that some fluorescently transplanted cells were negative for both neuronal and glial markers. The fate of these may include either mesenchymal cell types or undifferentiated precursors, as shown by others [28, 29]. Characterizing and optimizing temporal and spatial neuroglial differentiation in the host tissue is a critically important goal.

Notably, transplanted cells appeared to modulate the endogenous niche environment of germinal zones in the adult brain where increased endogenous neuronal differentiation was identified. While injury alone is able to stimulate endogenous neurogenesis [30] we observed significantly increased numbers of doublecortin + neurons in close proximity to transplanted ENSCs. Furthermore, while direct needle injection causes injury along the needle tract and could account for endogenous neurogenesis as a response to structural tissue damage, we observed a similar pattern of stimulated endogenous neurogenesis following *systemic* cell delivery in the concussion model (Fig. 3). In our models, the needle injury did not induce any notable increase in neurogenesis in non-transplanted controls and no increase in neurogenesis was observed along the needle tract. Furthermore, the area of neurogenesis was restricted to the site of transplanted ENSCs. Thus, the co-location of newly generated doublecortin-expressing endogenous neurons and the transplanted ENSCs is suggestive of cell transplantation associated endogenous neurogenesis, although this remains to be verified. Importantly, brain or whole body irradiation does not lead to any gross alteration in brain structure in the time periods we studied, although disruption of the functional integrity of the blood–brain barrier does occur [31]. Similarly, the concussion model employed does not lead to any gross alteration in brain structure [32].

In the present study we used a rodent allogenic transplant model, but our findings support the hypothesis that in humans, autologous gut-derived neural cells may offer a promising cell source for transplantation into the injured CNS. While ENSCs can be isolated from all intestinal segments and using donors of all ages [18, 33, 34], of particular clinical interest are mucosal/submucosal endoscopic biopsies as a strategy to harvest gut-derived progenitor cells. These techniques have been used to isolate and expand ENSc populations [35] and may provide a suitable source of cells derived from a minimally invasive intervention. Thus our data provide support for further studies to investigate whether small intestinal biopsies from adult donors can serve as a cell source for ENSCs transplantation. In order to validate the functional relevance of this proposed repair strategy, studies in disease-specific preclinical models are needed.

Our findings raise a number of questions that remain unanswered: What are the spatial and temporal dynamics of transplanted cells? What is the proportion of transplanted cells that survive and for how long? What is the extent of neuronal and glial differentiation in each model system, and what is the optimal timing for cell transplantation after injury to yield maximal benefit? What is the mechanism by which transplanted cells might stimulate brain plasticity (e.g., cell integration into the host network system and production of neuroprotective factors)? Perhaps most importantly, do transplanted cells result in functional recovery? While the current study was not designed to address these important questions or to provide quantitative data, our findings suggest that the use of autologous intestinal neural tissues for CNS repair might be a feasible strategy that warrants further investigation and could represent a promising avenue for rapid translation into the clinical arena.

## Additional file

10.1186/s12868-016-0238-y  Transplanted ENSCs can be identified in the lung 24 h post-delivery. Clusters of DsRed + cells (arrows) are identified in lung tissue 24 h following systemic delivery of ENSCs (boxed area magnified in inset).
