# Supplementary figures and images for: Engraftment of enteric neural progenitor cells into the injured adult brain
Source: BMC Neurosci. 2016 Jan 25;17:5. doi: 10.1186/s12868-016-0238-y (PMC4727306; doi:10.1186/s12868-016-0238-y)

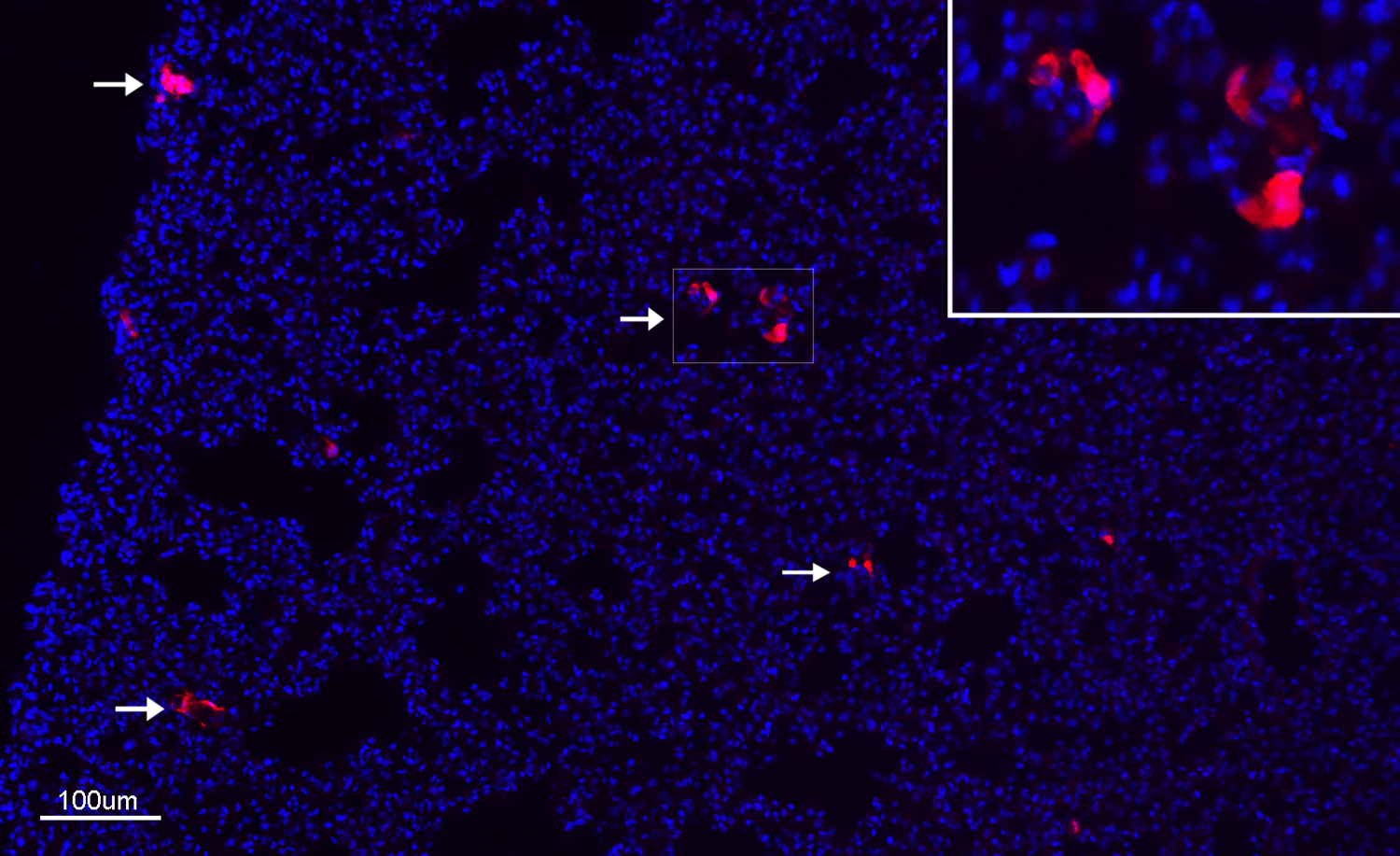

Supplement: Supplementary file 1 — 10.1186/s12868-016-0238-y Transplanted ENSCs can be identified in the lung 24 h post-delivery. Clusters of DsRed + cells (arrows) are identified in lung tissue 24 h following systemic delivery of ENSCs (boxed area magnified in inset). [file 12868_2016_238_MOESM1_ESM.tif]
